# Integrative analysis of TP53 mutations in lung adenocarcinoma for immunotherapies and prognosis

**DOI:** 10.1186/s12859-023-05268-2

**Published:** 2023-04-18

**Authors:** He Li, Lei Yang, Yuanyuan Wang, Lingchan Wang, Gang Chen, Li Zhang, Dongchang Wang

**Affiliations:** 1 Department of Respiration, The Third Hospital of Hebei Medical University, Shijiazhuang, China; 2 Department of Epidemiology and Statistics, School of Public Health, Hebei Key Laboratory of Environment and Human Health, Hebei Medical University, Shijiazhuang, China; 3 Department of Ultrasound, The Third Hospital of Hebei Medical University, Shijiazhuang, China; 4 Department of Geriatrics, The Third Hospital of Hebei Medical University, Shijiazhuang, China

**Keywords:** TP53, Lung adenocarcinoma, Tumor microenvironment, Signaling pathways, Immune checkpoint

## Abstract

**Background:**

The *TP53* tumor suppressor gene is one of the most mutated genes in lung adenocarcinoma (LUAD) and plays a vital role in regulating the occurrence and progression of cancer. We aimed to elucidate the association between *TP53* mutations, response to immunotherapies and the prognosis of LUAD.

**Methods:**

Genomic, transcriptomic, and clinical data of LUAD were downloaded from The Cancer Genome Atlas (TCGA) dataset. Gene ontology (GO) analysis, Kyoto Encyclopedia of Genes and Genomes (KEGG) enrichment analysis, gene set enrichment analysis (GSEA). Gene set variation analysis (GSVA) were performed to determine the differences in biological pathways. A merged protein–protein interaction (PPI) network was constructed and analyzed. MSIpred was used to analyze the correlation between the expression of the *TP53* gene, tumor mutation burden (TMB) and tumor microsatellite instability (MSI). CIBERSORT was used to calculate the abundance of immune cells. Univariate and multivariate Cox regression analyses were used to determine the prognostic value of *TP53* mutations in LUAD.

**Results:**

*TP53* was the most frequently mutated in LUAD, with a mutational frequency of 48%. GO and KEGG enrichment analysis, GSEA, and GSVA results showed a significant upregulation of several signaling pathways, including PI3K-AKT mTOR (P < 0.05), Notch (P < 0.05), E2F target (NES = 1.8, P < 0.05), and G2M checkpoint (NES = 1.7, P < 0.05). Moreover, we found a significant correlation between T cells, plasma cells, and *TP53* mutations (R^2^ < 0.01, P = 0.040). Univariate and multivariate Cox regression analyses revealed that the survival prognosis of LUAD patients was related to *TP53* mutations (Hazard Ratio (HR) = 0.72 [95% CI, 0.53 to 0.98], P < 0.05), cancer status (P < 0.05), and treatment outcomes (P < 0.05). Lastly, the Cox regression models showed that *TP53* exhibited good power in predicting three- and five-year survival rates.

**Conclusions:**

*TP53* may be an independent predictor of response to immunotherapy in LUAD, and patients with *TP53* mutations have higher immunogenicity and immune cell infiltration.

**Supplementary Information:**

The online version contains supplementary material available at 10.1186/s12859-023-05268-2.

## Introduction

Lung cancer is one of the deadliest malignancies, posing a major threat to human health. Its morbidity and mortality rates are increasing worldwide, in 2023; in 2023, it is anticipated that there will be 127,070 lung cancer deaths and 238,340 new cases [1]. Lung adenocarcinoma (LUAD), a main subtype of lung cancer, has displayed an increasing incidence rate, accounting for 38.5% of all lung cancer cases [2]. Owing to recent advances in research on molecular markers for the diagnosis and prognosis of LUAD, immunotherapies are currently applied in its treatment. Nevertheless, the prognosis of LUAD remains unfavorable and the survival rate of patients with LUAD has not improved. Therefore, it is crucial to identify molecular markers and understand the mechanisms by which these biomarkers affect treatment and prognosis.

*TP53*, a critical DNA repair gene, has been dubbed as a "guardian of the genome" [3, 4] that maintains the stability and integrity of genes. Mutated *TP53* leads to loss of tumor suppressor ability and accelerates tumor formation [5]. Although there is inadequate evidence to link *TP53* gene mutations to the immunobiological behavior and clinical features of lung cancer, there is evidence that *TP53* gene mutations to alter the sensitivity of immune checkpoint inhibitors (ICIs) treatment and resistance evolution of EGFR tyrosine kinase inhibitors in non-small cell lung cancer (NSCLS) [6–8]. *TP53* mutations have been reported to upregulate the expression of immune checkpoints, activate effector T cells, and affect a group of genes involved in cell cycle regulation, DNA replication, and damage repair in LUAD [8]. Sun et al. have demonstrated that a specific *TP53* mutation is a biomarker for checkpoint inhibitors in LUAD, and patients with LUAD harboring a *TP53* missense mutation show a superior response to immunotherapies [9]. Previous studies using bioinformatic analyses have identified several genes, including *TP53*, that are classified as effective prognostic markers and play critical roles in the initiation and progression of LUAD [10, 11]. However, the mechanism and clinical value of *TP53* as a possible biomarker in terms of multi-omics analysis (immunology, molecular biology, and genetics) and prognostic value are not yet investigated. Therefore, identification of the mechanisms that affect drug response and prognosis is critical for overcoming the therapeutic challenges associated with LUAD and accurately predicting its prognosis.

Bioinformatic analysis provides a comprehensive method for studying diverse multi-omics datasets. Therefore, in this study, we aimed to use bioinformatics and statistical analyses of data collected from patients with LUAD from The Cancer Genome Atlas (TCGA) dataset to determine the therapeutic and prognostic significance of *TP53* mutations. First, we searched gene expression profiling datasets for LUAD in TCGA to identify differences in gene expression. Second, we used the R package deconstruct Sigs to calculate tumor mutation burden (TMB) and microsatellite instability (MSI) in the mutated and wild-type *TP53* groups (TP53-MUT and TP53-WT, respectively). Third, we performed Gene Ontology (GO) and Kyoto Encyclopedia of Genes and Genomes (KEGG) pathway enrichment analyses, gene set enrichment analysis (GSEA), and gene set variation analysis (GSVA) to determine the differentially enriched signaling pathways. Furthermore, we constructed protein–protein interaction (PPI) networks and then used molecular complex detection (MCOD) to detect densely connected regions in these networks. Moreover, we applied ESTIMATE to quantify immunological activity in tumor samples and then calculated TP53-MUT and ESTIMATE score correlations. Finally, we created a prognostic model using clinicopathological features to predict the long-term survival rate of patients with LUAD, which was then used to construct a nomogram to guide clinical judgment. The workflow of our research is presented in Additional file 1: SF1.

## Results

### TP53 is the most frequently mutated gene in LUAD

**Fig. 1:**
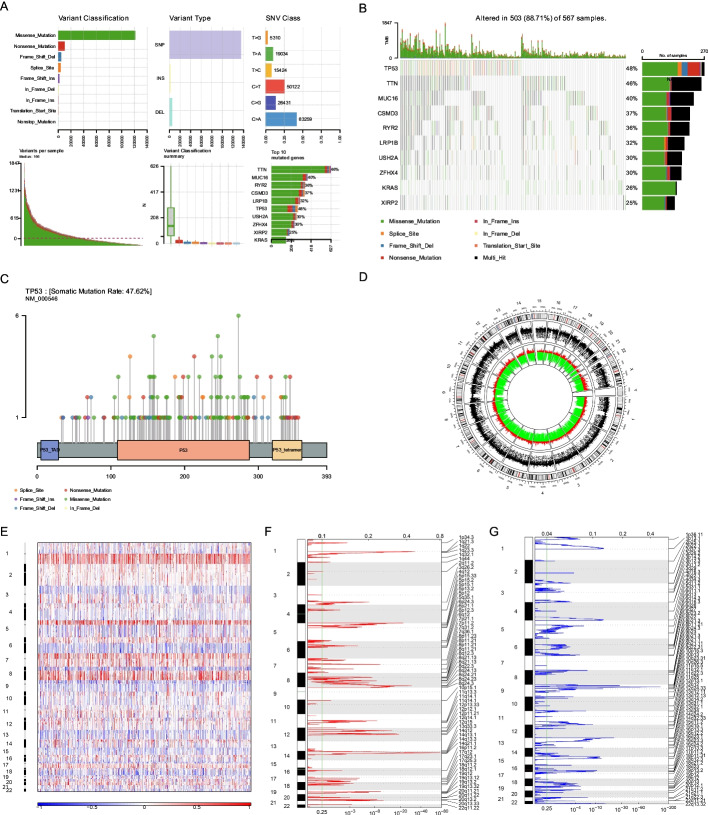
Analysis of copy number variation (CNV) and somatic mutation patterns of patients with lung adenocarcinoma (LUAD). **A** Mutation information statistic of LUAD patients in LUAD cohort of TCGA. **B** The top 10 most frequently mutated genes from LUAD patients in the cohort of TCGA. Left side of the panel shows the high mutation frequency genes in the waterfall plot, and the colors indicates different mutation types of the high mutation frequency genes in the right panel. (Genes are ordered by their mutation frequencies, and samples are ordered according to disease histology as indicated by the annotation bottom). **C** Lollipop plot displaying mutation distribution and protein domains for TP53 in LUAD with the labeled recurrent hotspots. Somatic mutation rate and transcript names are indicated by plot title and subtitle. **D** Schematic representation of the CNV in the TCGA-LUAD, the outermost ring represents the chromosomes, the red ring represents the gene expanded, and the green ring represents the gene deletion. **E–G** Identification of significantly differing gene amplifications and deletions. False discovery rates (Q-value) and score alteration of GISTIC2.0 (x axis) is plotted versus genome positions (y axis). The broken line represents centromeres. The green line represents the cut-off point of 0.25 Q for determining significance

We counted the number and frequency of different TP53 mutations in the top 10 (Additional file 2: ST1) and determined the most frequent types of mutations in patients with LUAD in the TCGA dataset (Fig. 1A). Of these, missense mutations were the most prevalent. We also determined the number of TCGA-LUAD-affected genes (Fig. 1B) and found that *TP53* (48%), *TTN* (46%), and *MUC16* (40%) had the highest mutational frequencies. Since *TP53* has the highest mutation frequency, we visualized its mutations. We found that most of the mutations in *TP53* were missense variants (Fig. 1C). To determine the copy number variation (CNV) and identify genes with substantial amplification or deletion, we used the CNV data from TCGA (Fig. 1D) and GISTIC 2.0, respectively. Our results showed that *TP53* did not exhibit any significant amplification or deletion (Fig. 1E–G). Therefore, *TP53* was incorporated as the biomarker for the treatment and prognosis of LUAD.

The Human Protein Atlas (HPA) database (https://www.proteinatlas.org/) is a free public database of over 26,000 antibodies targeting more than 17,000 human genes [12]. The immunohistochemical information on *TP53* was obtained from the HPA database and found to be significantly high-expression in normal and LUAD tissues, suggesting that *TP53* is a meaningful biomarker (Additional file 3: SF4).

### TP53 mutation and response to immunotherapy

**Fig. 2:**
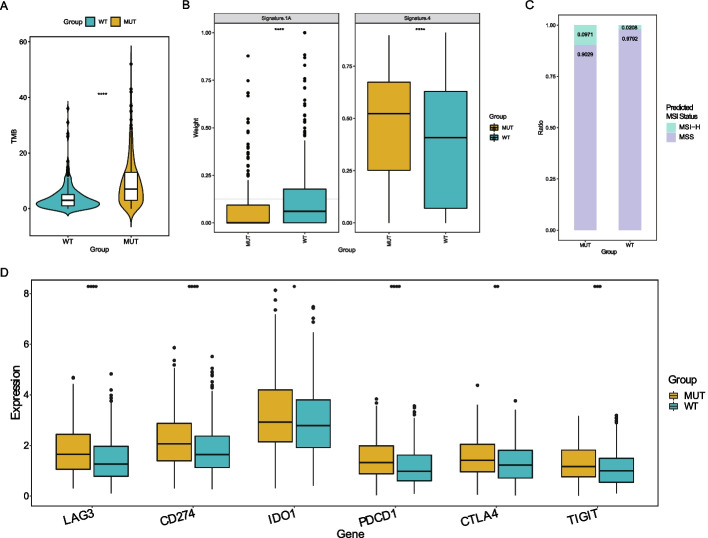
TP53 mutation and response to immunotherapy. **A**The TP53 mutation has a significance effect on TMB in lung adenocarcinoma patients. **B** The effect of TP53 mutation on the mutational signature. **C** The MSI status predicted by TP53 mutation grouping. **D** Difference of immune checkpoints expression on the TP53-MUT

We tallied the mutations in the TP53-MUT and TP53-WT groups and found that the TMB of the former was greater than that of the latter (P < 0.05), This suggests that the TP53-MUT group may be more responsive to immunotherapy (Fig. 2A).

Signature.nature2013 [13], a known signature inference of mutational differences between the mutant and wild-type groups, was selected to determine the relationship between TMB and treatment response. In the signature4 group, we found that TP53-MUT group was in the upper part of the boxplot, indicating a higher correlation with immunotherapy, but in the signature1A group, it was in lower part, indicating a poorer correlation (Fig. 2B). Based on the mutation data, we projected the state distribution of high microsatellite instability (MSI-H) and microsatellite stability (MSS) in the TP53-MUT and TP53-WT groups and found that the proportion of MSI-H in the TP53-MUT group was considerably greater than that in the TP53-WT group (the ratio of TP53-MUT group is 0.0971 and the ratio of TP53-WT group is 0.0208) (Fig. 2C). Therefore, we speculated that TP53-MUT samples would be more sensitive to immunotherapy and can thus benefit from immunotherapy. Simultaneously, we studied the variations in the expression of immune checkpoints between the TP53-MUT and TP53-WT groups (Fig. 2D). The expression of numerous common immune checkpoints, including *LAG3*, *IDO1*, *PDCD1*(*PD-1*), *CTLA4*, and *TIGIT*, was considerably higher in the TP53-MUT group than in the TP53-WT group (TP53-MUT group was in the upper part of the whole boxplot). This result suggests that TP53-MUT cells were more susceptible to ICIs.

### TP53 mutation and sensitivity to antineoplastic agents

**Fig. 3:**
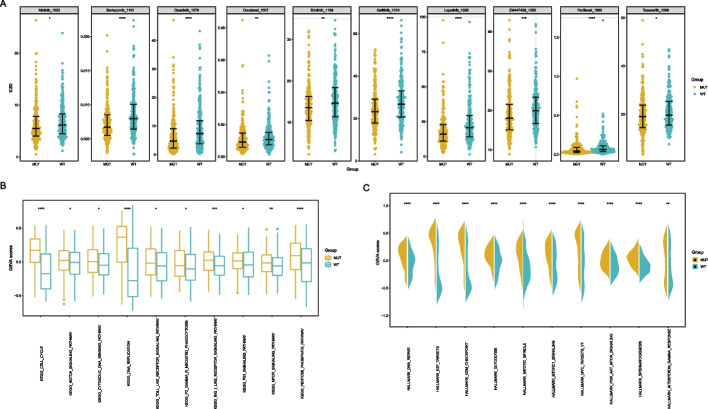
Analysis of drug sensitivity and differences in biological characteristics in patients with lung adenocarcinoma harboring mutations in TP53. **A** Difference in drug Sensitivity to LUAD with TP53-MUT and TP53-WT TP53, the horizontal axis is the TP53 mutation grouping, and the vertical axis is the 50% inhibitory concentration (IC50). **B** The difference of KEGG pathway between TP53-MUT and TP53-WT, the horizontal axis is the KEGG pathway, and the vertical axis is the signaling pathway enrichment score. **C** The difference of Hallmark between TP53-MUTand TP53-WT, the horizontal axis is hallmark, and the vertical axis is hallmark enrichment score

To further identify drugs that might interact with the TP53-MUT group of patients with LUAD, we determined the susceptibility of the TP53-MUT and TP53-WT groups to the currently available LUAD-specific therapies using gene expression data from the TCGA-LUAD dataset and drug sensitivity data from the GDSC database. The results indicated that individuals with *TP53* mutations were more susceptible to most medications with lower 50% inhibitory concentration (IC50) values, such as lapatinib, docetaxel, and erlotinib (Fig. 3A), all of which are often used in cancer treatment.

Furthermore, we calculated the influence of TP53-MUT on biological features and carcinogenic signaling pathways in TCGA-LUAD using GSVA. We found that cell cycle signaling and the Notch signaling pathway were upregulated in the TP53-MUT group (Fig. 3B). Additionally, the G2/M checkpoint and the PI3K-AKT-mTOR signaling pathway were markedly upregulated in the TP53-MUT group than in the TP53-WT group (Fig. 3C). Erlotinib may have a synergistic effect with *TP53* mutation in inhibiting the PI3K-AKT-mTOR signaling pathway, along with the significant upregulation of the latter in the TP53-MUT group, as shown above in the GSVA analysis.

### Functional characteristics of *TP53* mutations in LUAD

**Fig. 4:**
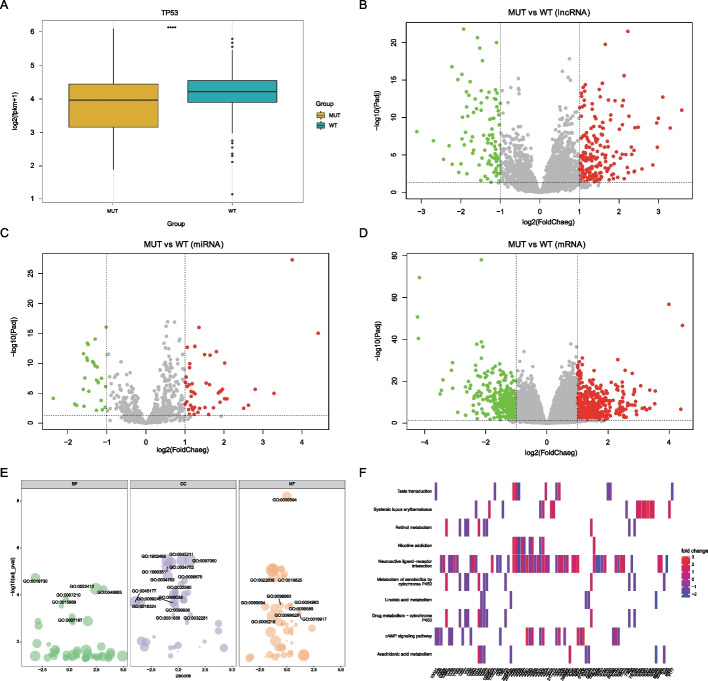
Differentially expressed genes analysis in mutated and wild-type TP3 groups in the cohort of patients with lung adenocarcinoma. **A** Association between the TP53 mutation and the TP53 expression. **B–D** Differential expression analysis. The horizontal axis is the log2 Fold Change, and the vertical axis is -log10(Adjust P value), Red nodes represent upregulation, blue node represent downregulation, and the gray node represents non-significant expression. B represents differentially expressed lncRNA, C represents differentially expressed miRNA, and D represents differentially expressed mRNA. **E** GO enrichment analysis was performed on differentially expressed mRNA. (F)KEGG pathway enrichment analysis

We examined the relationship between *TP53* mutations and gene expression (Fig. 4A) and found that *TP53* mutations may result in the signaling of gene expression. In addition, we identified 1298 differentially expressed genes (DEGs), including 277 differentially expressed lncRNAs and 837 differentially expressed mRNA, between the TP53-MUT and TP53-WT groups. The remaining 184 DEGs belonged to other gene types. In the TP53-MUT group, we identified 277 differentially expressed lncRNAs, of which 184 were upregulated (P < 0.05, logFC ≥ 1) and 93 were downregulated (P < 0.05, logFC ≤ − 1) (Fig. 4B), 74 differentially expressed miRNAs, including 47 upregulated (P < 0.05, logFC ≥ 1) and 27 downregulated miRNAs (P < 0.05, logFC ≤ − 1) (Fig. 4C), 837 differentially expressed mRNAs, of which 454 were upregulated (P < 0.05, logFC ≥ 1) and 383 were downregulated (P < 0.05, logFC ≤ − 1) (Fig. 4D).

To determine the functional characteristics of the upregulated and downregulated genes, we analyzed the biological processes, cell fractions, and molecular functions of 834 DEGs. First, we performed a functional enrichment analysis of GO (Fig. 4E, Table 1), which revealed that DEGs were significantly enriched in biological processes, such as antimicrobial humoral response, neuron fate commitment, cellular processes involved in reproduction in multicellular organisms, serotonin receptor signaling pathway, and gas transport. Several KEGG pathways (Fig. 4F, Table 2) enriched in our DEGs included genes involved in neuroactive ligand-receptor, nicotine addiction, drug metabolism-cytochrome P450, cAMP signaling pathway, xenobiotic metabolism by cytochrome P450, systemic lupus erythematosus, retinal metabolism, taste transduction, arachidonic acid metabolism, and linoleic acid metabolism.

**Table 1:** GO enrichment analysis

ONTOLOGY	ID	Description	P adjust
BP	GO:0019730	Antimicrobial humoral response	1.85E−05
BP	GO:0048663	Neuron fate commitment	5.55E−05
BP	GO:0022412	Cellular process involved in reproduction in multicellular organism	5.55E−05
BP	GO:0007210	Serotonin receptor signaling pathway	0.000151
BP	GO:0015669	Gas transport	0.000298
BP	GO:0007187	G protein-coupled receptor signaling pathway, coupled to cyclic nucleotide second messenger	0.000855
BP	GO:0003407	Neural retina development	0.001234
BP	GO:0019731	Antibacterial humoral response	0.001294
BP	GO:0007281	Germ cell development	0.001294
BP	GO:0098664	G protein-coupled serotonin receptor signaling pathway	0.001493
BP	GO:0007586	Digestion	0.002012
BP	GO:0042445	Hormone metabolic process	0.011584
BP	GO:0042403	Thyroid hormone metabolic process	0.012553
BP	GO:0021953	Central nervous system neuron differentiation	0.01564
BP	GO:0007188	Adenylate cyclase-modulating G protein-coupled receptor signaling pathway	0.020789
BP	GO:0007193	Adenylate cyclase-inhibiting G protein-coupled receptor signaling pathway	0.022382
BP	GO:0007286	Spermatid development	0.022382
BP	GO:0006590	Thyroid hormone generation	0.022394
BP	GO:0007389	Pattern specification process	0.022394
BP	GO:0042744	Hydrogen peroxide catabolic process	0.027606
BP	GO:0006323	DNA packaging	0.027619
BP	GO:0048515	Spermatid differentiation	0.027619
BP	GO:0009954	Proximal/distal pattern formation	0.029486
BP	GO:0015671	Oxygen transport	0.029486
BP	GO:0090596	Sensory organ morphogenesis	0.029486
BP	GO:0018958	Phenol-containing compound metabolic process	0.031485
BP	GO:0060078	Regulation of postsynaptic membrane potential	0.03358
BP	GO:0034508	Centromere complex assembly	0.034661
BP	GO:0042743	Hydrogen peroxide metabolic process	0.034661
BP	GO:0051321	Meiotic cell cycle	0.034661
BP	GO:0042391	Regulation of membrane potential	0.036642
BP	GO:0042737	Drug catabolic process	0.036642
BP	GO:0051932	Synaptic transmission, GABAergic	0.038723
BP	GO:0043486	Histone exchange	0.039256
BP	GO:0006959	Humoral immune response	0.039355
BP	GO:0000280	Nuclear division	0.039355
BP	GO:0140013	Meiotic nuclear division	0.041515
BP	GO:0060294	Cilium movement involved in cell motility	0.041769
BP	GO:0048562	Embryonic organ morphogenesis	0.041769
BP	GO:0007143	Female meiotic nuclear division	0.041769
BP	GO:0007214	Gamma-aminobutyric acid signaling pathway	0.041769
BP	GO:0035235	Ionotropic glutamate receptor signaling pathway	0.041769
BP	GO:0048665	Neuron fate specification	0.041769
…	…	…	…
MF	GO:0070330	Aromatase activity	0.040267
MF	GO:0004252	Serine-type endopeptidase activity	0.043303
MF	GO:0015079	Potassium ion transmembrane transporter activity	0.043303
MF	GO:0047498	Calcium-dependent phospholipase A2 activity	0.043303
MF	GO:0017171	Serine hydrolase activity	0.049034

**Table 2:** KEGG enrichment analysis

ID	Description	P adjust
hsa04080	Neuroactive ligand-receptor interaction	1.65E−13
hsa05033	Nicotine addiction	0.000143
hsa00982	Drug metabolism-cytochrome P450	0.000789
hsa04024	cAMP signaling pathway	0.001009
hsa00980	Metabolism of xenobiotics by cytochrome P450	0.001102
hsa05322	Systemic lupus erythematosus	0.001329
hsa00830	Retinol metabolism	0.005698
hsa04742	Taste transduction	0.008404
hsa00590	Arachidonic acid metabolism	0.009237
hsa00591	Linoleic acid metabolism	0.013333

**Fig. 5:**
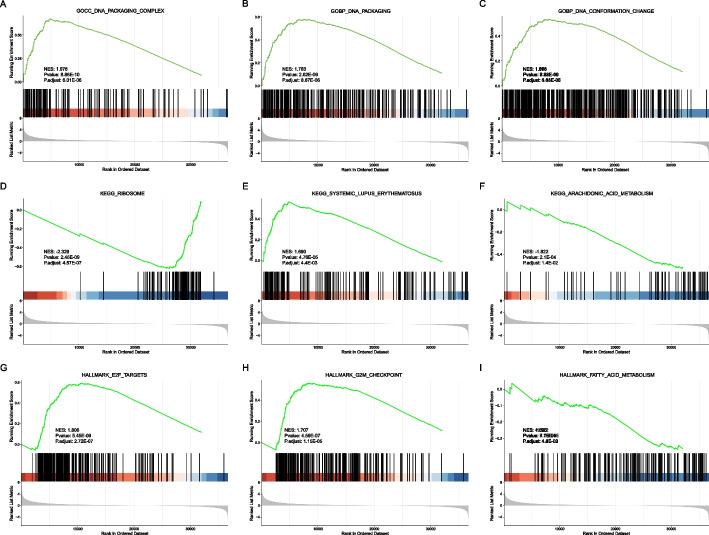
Gene set enrichment analysis (GSEA) function enrichment analysis. **A–G** Results of GSEA enrichment analysis. **A–C** Top 3 GO enrichments. **D–F** Top 3 KEGG pathway enrichments. **G–I** Top 3 Hallmark pathway enrichments

We also analyzed the biological function enrichment of TP53-MUT and TP53-WT genes in GSEA (Fig. 5, Table 3). The results demonstrated that genes in the TP53-MUT and TP53-WT groups were largely enriched in the DNA packaging complex, GOBP DNA packaging, GOBP DNA conformation change, and other GO pathways (Fig. 5A–C). KEGG functional pathways were enriched in ribosomes, systemic lupus erythematosus, and arachidonic acid metabolism. (Fig. 5D–F). Cancer-related pathways were enriched in Hallmark-E2f-targets, Hallmark-G2M-checkpoint, Hallmark-fatty-acid-metabolism, and Hallmark-Spermatogenesis (Fig. 5G-I). Based on the results, we assumed that DEGs may regulate the metabolic pathways, such as ribosomes, arachidonic acid metabolism, Hallmark-E2f-targets, and Hallmark-G2M-checkpoint.

**Table 3:** GSEA enrichment analysis

ID	NES	P value	P adjust
*GSEA GO*			
GOCC_DNA_PACKAGING_COMPLEX	1.976383	8.88E−10	6.01E−06
GOBP_DNA_PACKAGING	1.783477	2.62E−09	8.87E−06
GOBP_DNA_CONFORMATION_CHANGE	1.668193	7.22E−09	1.48E−05
GOBP_NUCLEOSOME_ASSEMBLY	1.902487	8.74E−09	1.48E−05
GOBP_NUCLEOSOME_ORGANIZATION	1.81451	1.28E−08	1.74E−05
GOBP_CHROMATIN_ASSEMBLY_OR_DISASSEMBLY	1.732312	6.18E−08	6.97E−05
GOBP_CHROMOSOME_SEGREGATION	1.616316	8.28E−08	8.01E−05
GOBP_NEGATIVE_REGULATION_OF_GENE_EXPRESSION_EPIGENETIC	1.837812	1.76E−07	0.000149
GOCC_PROTEIN_DNA_COMPLEX	1.723961	2.38E−07	0.000179
GOBP_ORGANELLE_FISSION	1.518382	3.71E−07	0.000232
GOBP_PROTEIN_DNA_COMPLEX_SUBUNIT_ORGANIZATION	1.655031	3.77E−07	0.000232
GOCC_NUCLEAR_CHROMOSOME	1.660653	5.66E−07	0.000315
GOBP_MEIOTIC_CELL_CYCLE_PROCESS	1.712118	6.30E−07	0.000315
GOBP_NUCLEAR_CHROMOSOME_SEGREGATION	1.63877	6.51E−07	0.000315
GOCC_MULTIVESICULAR_BODY	−2.26426	9.31E−07	0.00042
GOBP_CHROMATIN_ORGANIZATION_INVOLVED_IN_REGULATION_OF_TRANSCRIPTION	1.748391	1.08E−06	0.000458
GOCC_CHROMOSOMAL_REGION	1.559684	1.23E−06	0.00049
GOBP_MEIOTIC_CELL_CYCLE	1.642938	1.45E−06	0.000544
GOBP_NEURON_FATE_COMMITMENT	1.902468	2.48E−06	0.000882
GOMF_SERINE_HYDROLASE_ACTIVITY	−1.74924	2.97E−06	0.001006
GOCC_CONDENSED_CHROMOSOME	1.644496	3.86E−06	0.001245
GOCC_CYTOSOLIC_RIBOSOME	−1.93214	4.94E−06	0.001518
GOBP_MITOTIC_NUCLEAR_DIVISION	1.559838	1.16E−05	0.00342
GOBP_MEIOSIS_I_CELL_CYCLE_PROCESS	1.723002	1.29E−05	0.003639
GOBP_DOUBLE_STRAND_BREAK_REPAIR	1.536981	2.65E−05	0.006998
GOBP_REGULATION_OF_GENE_EXPRESSION_EPIGENETIC	1.594629	2.69E−05	0.006998
GOBP_CHROMOSOME_ORGANIZATION_INVOLVED_IN_MEIOTIC_CELL_CYCLE	1.792989	2.87E−05	0.007197
GOBP_MITOTIC_SISTER_CHROMATID_SEGREGATION	1.653645	3.52E−05	0.008504
GOBP_REGULATION_OF_NUCLEAR_DIVISION	1.686256	3.66E−05	0.008541
GOBP_POSITIVE_REGULATION_OF_LYMPHOCYTE_APOPTOTIC_PROCESS	1.88292	4.13E−05	0.009313
GOCC_CILIARY_PLASM	−1.74751	5.04E−05	0.010778
GOCC_BRUSH_BORDER_MEMBRANE	−2.01561	5.23E−05	0.010778
GOCC_DENSE_CORE_GRANULE	1.84658	5.38E−05	0.010778
GOCC_CHROMOSOME_CENTROMERIC_REGION	1.558626	5.42E−05	0.010778
GOBP_MEIOTIC_CHROMOSOME_SEGREGATION	1.732776	5.70E−05	0.011016
GOCC_CONDENSED_NUCLEAR_CHROMOSOME	1.724235	6.16E−05	0.01129
GOBP_SISTER_CHROMATID_SEGREGATION	1.580149	6.22E−05	0.01129
GOBP_CILIUM_MOVEMENT	−1.61535	6.34E−05	0.01129
GOBP_FLUID_TRANSPORT	−2.16657	7.20E−05	0.012489
GOMF_CALCIUM_DEPENDENT_CYSTEINE_TYPE_ENDOPEPTIDASE_ACTIVITY	−2.11512	8.01E−05	0.013254
GOBP_CHROMATIN_SILENCING	1.756168	8.03E−05	0.013254
GOBP_AXONEME_ASSEMBLY	−1.8582	9.71E−05	0.015641
GOBP_HYDROGEN_PEROXIDE_BIOSYNTHETIC_PROCESS	−2.10282	9.95E−05	0.015653
GOCC_BRUSH_BORDER	−1.74031	0.000108	0.016545
GOMF_PEPTIDASE_REGULATOR_ACTIVITY	−1.53378	0.000116	0.017381
GOBP_DNA_DEPENDENT_DNA_REPLICATION	1.588182	0.000119	0.017563
GOBP_CELL_CYCLE_DNA_REPLICATION	1.751897	0.000122	0.017604
GOBP_VASCULAR_PROCESS_IN_CIRCULATORY_SYSTEM	−1.55854	0.000127	0.017821
GOCC_NEURONAL_DENSE_CORE_VESICLE	1.815389	0.000131	0.017821
GOBP_REGULATION_OF_MITOTIC_NUCLEAR_DIVISION	1.662135	0.000134	0.017821
GOBP_POSITIVE_REGULATION_OF_T_CELL_APOPTOTIC_PROCESS	1.813644	0.000134	0.017821
GOCC_BASAL_PART_OF_CELL	−1.52966	0.000145	0.01858
GOMF_ENDOPEPTIDASE_REGULATOR_ACTIVITY	−1.53689	0.000146	0.01858
GOBP_CENTROMERE_COMPLEX_ASSEMBLY	1.746264	0.00018	0.022575
GOBP_DNA_REPLICATION	1.470602	0.000195	0.023955
GOBP_RDNA_HETEROCHROMATIN_ASSEMBLY	1.781571	0.000205	0.024659
GOMF_SERINE_TYPE_ENDOPEPTIDASE_INHIBITOR_ACTIVITY	−1.74739	0.000208	0.024659
GOBP_HYPOTHALAMUS_DEVELOPMENT	1.816552	0.000235	0.026771
GOBP_SENSORY_PERCEPTION_OF_SMELL	1.412688	0.000235	0.026771
GOBP_EPITHELIAL_STRUCTURE_MAINTENANCE	−2.07214	0.000237	0.026771
GOBP_WATER_TRANSPORT	−2.08751	0.000252	0.027924
GOBP_LIPID_OXIDATION	−1.65664	0.000313	0.0342
GOMF_HORMONE_ACTIVITY	1.60724	0.000329	0.035296
GOBP_HISTONE_EXCHANGE	1.722975	0.00034	0.035964
GOBP_HOMOLOGOUS_CHROMOSOME_SEGREGATION	1.7319	0.000353	0.036167
GOBP_MICROTUBULE_BUNDLE_FORMATION	−1.68493	0.000353	0.036167
GOBP_SENSORY_PERCEPTION_OF_CHEMICAL_STIMULUS	1.364093	0.000405	0.04081
GOBP_MAINTENANCE_OF_GASTROINTESTINAL_EPITHELIUM	−2.02779	0.000413	0.04081
GOCC_MICROVILLUS	−1.69866	0.000423	0.04081
GOBP_CELLULAR_MODIFIED_AMINO_ACID_METABOLIC_PROCESS	−1.46584	0.000424	0.04081
GOBP_POSITIVE_REGULATION_OF_LEUKOCYTE_APOPTOTIC_PROCESS	1.788148	0.000428	0.04081
GOMF_OXIDOREDUCTASE_ACTIVITY_ACTING_ON_CH_OH_GROUP_OF_DONORS	−1.57945	0.000451	0.042352
GOMF_OLFACTORY_RECEPTOR_ACTIVITY	1.405798	0.000461	0.042709
GOMF_WATER_TRANSMEMBRANE_TRANSPORTER_ACTIVITY	−2.05169	0.000547	0.049989
*GSEA KEGG*			
KEGG_RIBOSOME	−2.32914	2.45E−09	4.57E−07
KEGG_SYSTEMIC_LUPUS_ERYTHEMATOSUS	1.690071	4.76E−05	0.004424
KEGG_ARACHIDONIC_ACID_METABOLISM	−1.82198	0.000218	0.013522
KEGG_LINOLEIC_ACID_METABOLISM	−1.93402	0.000932	0.033491
KEGG_VASCULAR_SMOOTH_MUSCLE_CONTRACTION	−1.54076	0.001054	0.033491
KEGG_COMPLEMENT_AND_COAGULATION_CASCADES	−1.70657	0.00108	0.033491
KEGG_FATTY_ACID_METABOLISM	−1.75378	0.001699	0.045149
*GSEA HALLMARK*			
HALLMARK_E2F_TARGETS	1.806064	5.45E−09	2.72E−07
HALLMARK_G2M_CHECKPOINT	1.70655	4.59E−07	1.15E−05
HALLMARK_FATTY_ACID_METABOLISM	−1.52192	0.000714	0.011892
HALLMARK_SPERMATOGENESIS	1.507066	0.002415	0.030192

### Construction of protein–protein interaction (PPI) networks

**Fig. 6:**
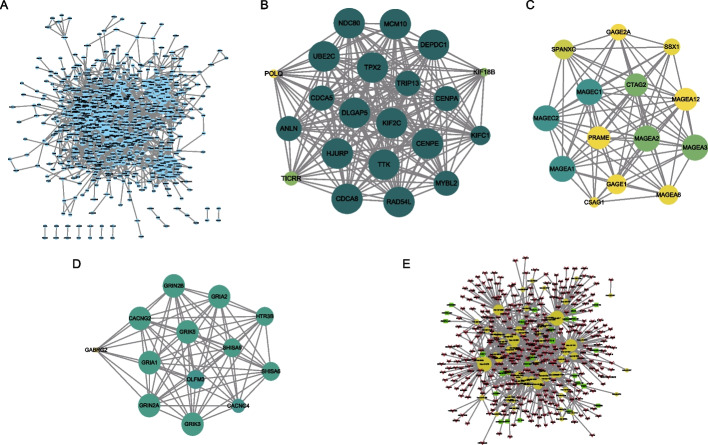
Protein–protein interaction network analysis. **A** Protein–protein intersection network of differentially expressed genes in TP53-MUT and TP53-WT patients. Node size represents the degree of connectivity of the indicated protein in the network. **B** The sub-network module 1 in PPI. Color node denote the MOCDE score for the module and node size represent the degree of connectivity of the module. **C** The sub-network module 2 in PPI. Color node denote the MOCDE score for the module and node size represent the degree of connectivity of the module. **D** The sub-network module 3 in PPI. Color node denote the MOCDE score for the module and node size represent the degree of connectivity of the module. **E** ceRNA (mRNA-miRNA-lncRNA) network. Yellow dots indicate miRNA and red arrows indicate mRNA, whereas green rectangles indicate lncRNA

We constructed PPI networks based on 814 DEGs between TP53-MUT and TP53-WT groups (Fig. 6A) which included 2767 PPIs. The average degree of nodes was 6.8 and PPI enrichment had P < 1.0e^−16^. Three subnet modules in the DEG-PPI network were discovered using multicontrast delayed enhancement (MCODE). The first module was separated into 20.1, which included 21 gene nodes (Fig. 6B); the second module was divided into 12.462, which included 14 gene nodes (Fig. 6C); and the third module was divided into 12.167, which included 13 gene nodes (Fig. 6D). These PPI network construction results demonstrated that the gene nodes were closely associated with tumor metabolism, e.g., *KIFC1* may contribute to the movement of early endocytic vesicles and regulates cilium formation and tumorigenesis.

We identified differentially expressed mRNAs (deg-mRNA), miRNAs (deg-miRNA), and lncRNAs (deg-lncRNA) between TP53-MUT and TP53-WT groups and acquired mRNAs (target-mRNA) and lncRNA (target-lncRNA) sequences matching the deg-miRNA in the miRNet database. Next, we took the intersected of deg-mRNA and target-mRNA, the intersection of deg-lncRNA and target-lncRNA. And then we used these two intersections to construct the mRNA-miRNA-lncRNA network. The intersecting mRNA-miRNA-lncRNA network revealed 765 interactions among 327 mRNAs, 60 miRNAs, and 26 lncRNAs (Fig. 6E). The high interactions of all these mRNAs, miRNAs, and lncRNAs were associated with suppression of cell multiplication and induced apoptosis in TP53-MUT group.

### Immune infiltration analysis

**Fig. 7:**
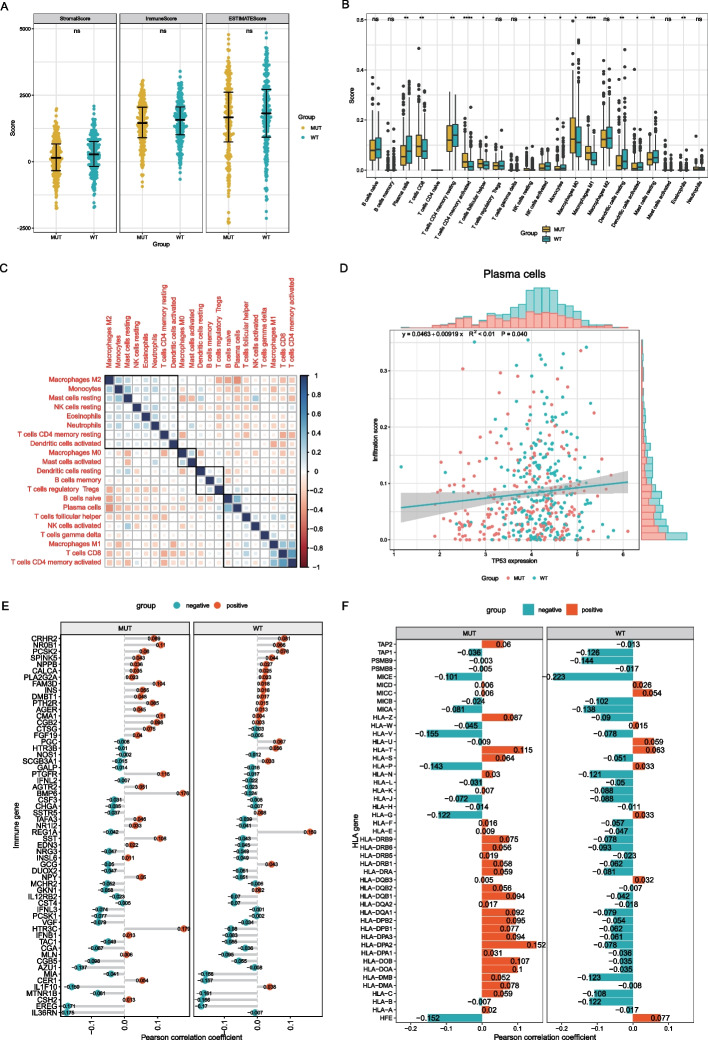
TP53 mutation and tumor infiltrates immune cells (TIICs). **A** Overall immune infiltration in the TP53-MUT and the TP53-WT. **B** Immune cell content in TP53-MUT and TP53-WT group. The horizontal axis is the immune cell, the vertical axis is the immune cell content. **C** Immunocyte-associated Heatmap. Blue is positive correlation and red is negative correlation. **D** Association between TP53-MUT and Plasma cell. **E** Association between TP53-MUT and the immune gene. **F** Association between the family of HLA gene

The total immune infiltration of the TP53-MUT and TP53-WT cohorts was analyzed. We compared the stromal and immune scores of the two groups and found no statistically significant difference in immune infiltration between the two groups (Fig. 7A). Next, we compared and analyzed the number of tumor-infiltrating immune cells (TIICs) between the two groups. The results indicated that the number of several immune cells, such as CD8, CD4 memory cells, and T follicular helper cells were considerably greater in the TP53-MUT group than in the TP53-WT group (Fig. 7B, Additional file 4: SF3). We examined the associations between immune cells in the TP53-MUT group and discovered that most immune cells had negative correlations (Fig. 7C). We used correlation curve fitting to analyze *TP53* expression and infiltration of different immune cells and found that *TP53* expression was significantly correlated with plasma cell infiltration (Fig. 7D).

In addition, we evaluated the correlation between immune-related genes, *HLA* family genes, and *TP53* mutations. The results (Fig. 7E) showed negative correlation patterns between *PGC*, *HTR38*, *NOS1*, and *GALP* genes in the TP53-MUT and TP53-WT groups. For *HLA* genes (Fig. 7F), the correlation patterns between typical *HLA* genes in the two groups were mostly negative, whereas those of atypical *HLA* genes were mostly positive. The above results indicated that *TP53* mutations might upregulate the expression of immune response genes without significantly impacting immune stromal cells or the related genes.

### Construction and evaluation of the prognostic model

**Fig. 8:**
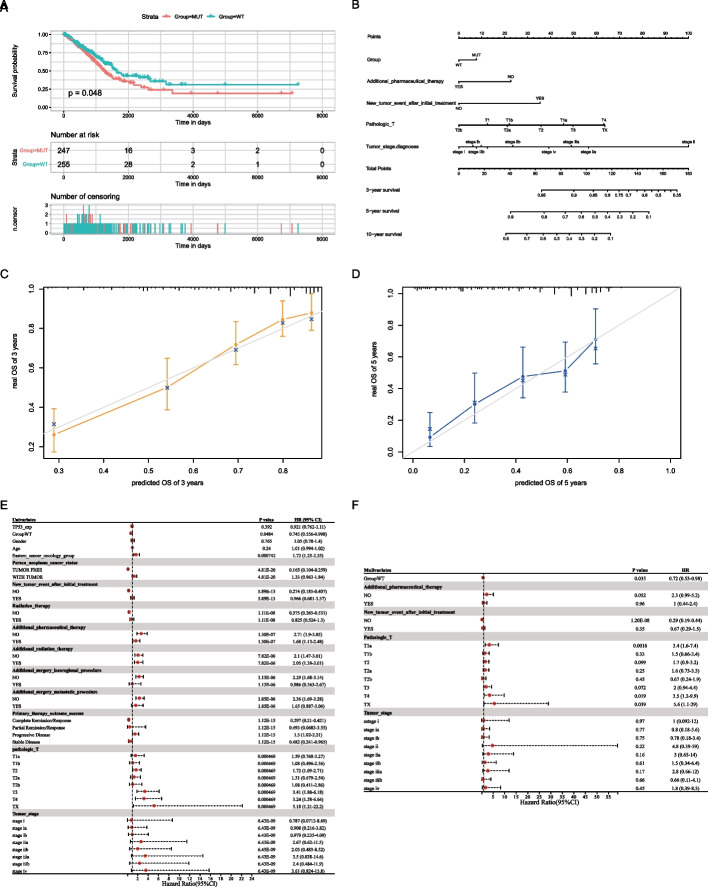
Construction and validation of a prognostic model in lung adenocarcinoma (LUAD). **A** Survival analysis of TP53 mutation. **B** Nomogram. **C, D** Prediction curve of 3-year survival and 5-year survival of LUAD patients with NOMO model. **E** Univariate COX Analysis. **F** Multivariate COX Analysis

Owing to the strong association between *TP53* and immunotherapy, we directly analyzed the effect of TP53-MUT on prognosis, and the results (Fig. 8A) indicated that the TP53-MUT group had a poor prognosis. Therefore, we further analyzed variables influencing the prognosis, such as TP53-MUT data of patients with LUAD, *TP53* expression data, and patient age, sex, clinical staging, and tumor staging in univariate and multivariate Cox regression analyses. Univariate Cox regression analyses revealed that age, gender, and *TP53* expression (TP53-exp) had no effect on prognosis, whereas *TP53* mutations, cancer status, and treatment outcomes affected the prognosis of patients with LUAD (Fig. 8E and Table 4; Age, HR = 1.01 [95% CI, 0.994 to 1.02], P = 0.24; Gender, HR = 1.05 [95% CI, 0.78 to 1.4], P = 0.765; TP53-exp, HR = 0.921 [95% CI, 0.762 to 1.11], P = 0.392; TP53-WT, HR = 0.745 [95% CI, 0.556 to 0.998], P < 0.05; additional therapy, P < 0.05; cancer status, P < 0.05). According to the results of the multivariate Cox regression analysis (Fig. 8F, Table 5), *TP53* mutations, cancer status, and new tumor events after initial treatment affected the prognosis (TP53-WT, HR = 0.72 [95% CI, 0.53 to 0.98], P < 0.05; new tumor event after initial treatment, P < 0.05; cancer status, P < 0.05). We included these indicators in a prognostic model, created a clinical prediction line chart (Fig. 8B), and assessed the model's predictive ability. The results showed that this model is highly predictive of the 3-, 5-, and 10-year survival rates of patients with LUAD. The model was calibrated, and the calibration curve indicated that the 3- and 5-year predictive values of the model were strong (Fig. 8C–D).

**Table 4:** Univariate and multivariate Cox regression analysis of TP53 gene mutation combined with clinicopathologic features

	HR (95% CI)	P value
*Univariates*		
TP53-exp	0.921 (0.762–1.11)	0.392
Group-WT	0.745 (0.556–0.998)	0.0484
Gender	1.05 (0.78–1.4)	0.765
Age	1.01 (0.994–1.02)	0.24
Eastern cancer oncology group	1.72 (1.25–2.35)	0.000742
Person neoplasm cancer status		
TUMOR FREE	0.165 (0.104–0.259)	4.81E−20
WITH TUMOR	1.33 (0.963–1.84)	4.81E−20
New tumor event after initial treatment		
NO	0.274 (0.185–0.407)	5.89E−13
YES	0.966 (0.681–1.37)	5.89E−13
Radiation therapy		
NO	0.375 (0.265–0.531)	1.11E−08
YES	0.825 (0.524–1.3)	1.11E−08
Additional pharmaceutical therapy		
NO	2.71 (1.9–3.85)	1.30E−07
YES	1.68 (1.13–2.48)	1.30E−07
Additional radiation therapy		
NO	2.1 (1.47–3.01)	7.82E−06
YES	2.05 (1.39–3.01)	7.82E−06
Additional surgery locoregional procedure		
NO	2.29 (1.68–3.14)	1.13E−06
YES	0.986 (0.363–2.67)	1.13E−06
Additional surgery metastatic procedure		
NO	2.36 (1.69–3.28)	1.85E−06
YES	1.65 (0.887–3.06)	1.85E−06
Primary therapy outcome success		
Complete Remission/Response	0.297 (0.21–0.421)	1.12E−15
Partial Remission/Response	0.493 (0.0683–3.55)	1.12E−15
Progressive Disease	1.5 (1.02–2.21)	1.12E−15
Stable Disease	0.482 (0.241–0.965)	1.12E−15
Pathologic-T		
T1a	1.59 (0.768–3.27)	0.000469
T1b	1.08 (0.496–2.36)	0.000469
T2	1.72 (1.09–2.71)	0.000469
T2a	1.31 (0.679–2.54)	0.000469
T2b	1.08 (0.411–2.86)	0.000469
T3	3.41 (1.88–6.18)	0.000469
T4	3.24 (1.58–6.64)	0.000469
TX	5.18 (1.21–22.2)	0.000469
Tumor stage		
Stage	0.787 (0.0712–8.69)	6.43E−09
Stage IA	0.908 (0.216–3.82)	6.43E−09
Stage IB	0.979 (0.235–4.09)	6.43E−09
Stage IIA	2.67 (0.62–11.5)	6.43E−09
Stage IIB	2.03 (0.483–8.52)	6.43E−09
Stage IIIA	3.5 (0.838–14.6)	6.43E−09
Stage IIIB	2.4 (0.484–11.9)	6.43E−09
Stage IV	3.61 (0.824–15.8)	6.43E−09
*Multivariates*		
Group-WT	0.72 (0.53–0.98)	0.035
Additional pharmaceutical therapy		
NO	2.3 (0.99–5.2)	0.052
YES	1 (0.44–2.4)	0.96
New tumor event after initial treatment		
NO	0.29 (0.19–0.44)	1.20E−08
YES	0.67 (0.29–1.5)	0.35
Pathologic T		
T1a	3.4 (1.6–7.4)	0.0016
T1b	1.5 (0.66–3.4)	0.33
T2	1.7 (0.9–3.2)	0.099
T2a	1.6 (0.73–3.3)	0.25
T2b	0.67 (0.24–1.9)	0.45
T3	2 (0.94–4.4)	0.072
T4	3.5 (1.2–9.9)	0.019
TX	5.6 (1.1–29)	0.039
Tumor stage		
Stage I	1 (0.092–12)	0.97
Stage IA	0.8 (0.18–3.6)	0.77
Stage IB	0.78 (0.18–3.4)	0.75
Stage II	4.8 (0.39–59)	0.22
Stage IIA	3 (0.65–14)	0.16
Stage IIB	1.5 (0.34–6.4)	0.61
Stage IIIA	2.8 (0.66–12)	0.17
Stage IIIB	0.66 (0.11–4.1)	0.66
Stage IV	1.8 (0.39–8.3)	0.45

## Discussion

LUAD is associated with a high mortality rate. Although the mortality rate of lung cancer is decreasing annually, current treatment and prognosis are not promising [14]. Owing to the high heterogeneity of LUAD, treatment modalities such as small-molecule targeted therapy and immunotherapy have limitations [15], such as sensitivity of tumor cells to various target drugs, the degree of drug resistance, and the target for antitumor immunotherapy. Hence, there is an urgent need to discover new immune checkpoints and treatment targets and understand the relationship between biomarkers and LUAD development and growth. Recently, several studies have suggested that *TP53* mutations in combination with mutations in *KRAS*, *EGFR* and *STK11* could affect the efficacy of ICIs, which makes *TP53* gene a good candidate for newly biomarkers. In this study, first we validated that *TP53* confers a high immunogenicity of tumors by calculating the association between *TP53* mutations, TMB, and MSI. Based on these calculations, we then investigated which *TP53* mutations affect the key immune checkpoints and demonstrated that *TP53* mutations may upregulate the expression of immunological checkpoints including *CTLA4* and *PDCD1*. Secondly, we analyzed the correlation between *TP53* mutation and targeted drug sensitivity. We found that the TP53-MUT group showed a lower IC50 for drugs such as erlotinib. We further analyzed the association between *TP53* mutations and inhibition of metabolic pathways by drugs. We found that *TP53* mutations have synergistic effects with targeted drugs in inhibiting tumor metabolism. Third, on the basis of the regulation of *TP53* on metabolic pathways, we performed GO and KEGG enrichment analyses, and the results showed that the PI3K-AKT-mTOR signaling pathway was regulated. Then, we constructed PPI networks to identify hub genes such as KIFCI that are related to the inhibition metabolism of LUAD. Finally, by investigating the TIICs and tumor immune response genes in the TP53-MUT group, we found that *TP53* mutations may upregulate the expression of HLA and increase TIICs to improve the immune response of patients with LUAD.

In summary, using bioinformatics analysis, we identified *TP53* as a key gene with good prognostic and therapeutic values in LUAD and suggested three mechanisms. First, *TP53* mutations increased the responsiveness of LUAD patients to ICIs by upregulating the expression of immune checkpoints. Second, *TP53* gene mutations increased the sensitivity of LUAD patients to antineoplastic drugs and reduced the risk of LUAD progression by upregulating the expression of the PI3K-AKT-mTOR pathway and G2/M checkpoint signaling. Thirdly, *TP53* mutations improved the immune response of patients with LUAD, by upregulating the expression of *HLA*. Some studies have demonstrated that higher TMB and MSI in LUAD would probably induce a potent immunogenic neoantigen that improves the response to immune checkpoint inhibitor treatments [16–18]. A previous study has reported the effect of *TP53* mutations on the immune checkpoints of patients with LUAD [19]. Additionally, Biton et al. showed that *TP53* mutations predict the response of patients with LUAD to anti-PD-1 through lymphocyte infiltration in the tumor immune microenvironment (TIME) [20]. However, the above studies had limitations in that they lacked the multi-omics approach on the mechanism and clinical prognostic value of *TP53* in LUAD. LncRNAs regulated by the *TP53* gene play crucial roles in the expression of immune checkpoints [21]. Additionally, a previous study has shown that *TP53* regulates programmed cell death 1 ligand 1 (PDL1) via miR-34 and that the immune checkpoints of tumor cells were transcriptionally regulated [22]. In this study, we first explored the role of *TP53* mutations in LUAD by calculating the TMB and MSI between the TP53-MUT and TP53-WT groups of a cohort of patients with LUAD. We found that the TP53-MUT group had higher TMB and MSI values. We also found that the TP53-MUT group may upregulate the expression *PDCD1* and *CTLA4*. Next, we constructed PPI networks to verify the result.

We also found that by regulating biological signaling pathways, *TP53* may affect the response to immunotherapy and the prognosis of LAUD. In our study, the results of GO and KEGG enrichment analyses on DEGs showed that the metabolic pathways of tumor progress were significantly enriched in the TP53-MUT, which verified *TP53* mutations can prevent progression of LUAD. A previous study has demonstrated that TP53-regulated downstream pathways, such as PI3K-AKT-mTOR, can alter immune responses by inducing a shift in anabolism, which is essential for T lymphocytes [23]. *TP53* encodes a transcription factor that plays an important role in the cell cycle. DNA damage or mutations in oncogenes, such as *Ras* and *MYC*, induce the activation of p53, leading to the activation of the tumor suppressor p21, which in turn inhibits tumor formation. The p53 blocks the G2/M checkpoint, which is the last barrier preventing damaged DNA from entering the mitotic phase by regulating the expression of cyclin B. Cyclin B can form a complex with cyclin B (Cdc2[Cdk1]-cyclin B), which is a key factor in G2/M checkpoint regulation [24]. Additionally, p53 is highly expressed in lung cancer. Collectively, these findings support the notion that mutated *TP53* is a potential target for signaling pathway suppression. Overall, these results are consistent with our findings of upregulated expression of pathways in the GO and KEGG enrichment analyses.

We were also interested in the relationship between *TP53* mutations and the TIME. In terms of tumor, we discovered that *PGC* and *HLA* downregulated and upregulated immune genes respectively. The PGC is a co-inhibitory molecule in the T cells. A study suggested that PGC1α-overexpressing T cells may appear exhausted as a result of loss of mitochondrial capacity and glucose metabolism defects [25]. *HLA* is a crucial immune gene, and the antigen-presenting HLA class I and II molecules are fundamental for triggering anti-tumor immunity. Another study suggested that when *HLA* expression is strong, high *HLA* allelic diversity may help more with tumor eradication by presenting a varied pool of neoantigens [26]. Immunotherapy for LUAD relies primarily on the role of T lymphocytes, which depends on their surface receptors to bind to the antigens presented by HLA molecules on the tumor cell surface. Based on the idea that *TP53* has a potential effect on the TIME and enhances the immune response [27]. We analyzed the expression of immune genes with TP53-MUT and suggested that *TP53* mutations regulate the TIME via upregulating expression of *HLA* and downregulating *PGC*. Additionally, previous studies have demonstrated that CD8 and CD4 T cell infiltration activation is not only the basis of tumor immunotherapy but also a prognostic indicator of whether the patient is responsive to immunotherapeutic agents [28]. In our study, we found that T cells CD4 memory and plasma cells were comparatively upregulated in the TP53-MUT group.

Our study, however, has some limitations. First, we simply discussed the potential of *TP53* as a new therapeutic target and did not perform a thorough analysis of functional enrichment or loss in the *TP53* mutation. Second, while we evaluated the prognosis of the TP53-MUT and TP53-WT groups, the prognostic analysis of the TP53-MUT group could not distinguish between different therapies. However, varying treatments may have different outcomes, and the different *TP53* mutation types of LUAD have a variable prognosis. Finally, our study has not been confirmed in further experiment. Nevertheless, our study provides valuable information and insights for future LUAD research.

## Conclusion

In conclusion, our study demonstrated the immunotherapeutic and prognostic value of *TP53* in patients with LUAD. These findings were used to elucidate the mechanism through which *TP53* mutations enhance the response to immunotherapy and helped construct a prognostic model to effectively predict the overall survival of patients with LUAD. We would like to perform more careful examinations of the diagnosis and treatment effects of TP53 by combining in vivo and in vitro techniques, which will be conducive to the development of novel techniques targeting *TP53* for the treatment of LUAD in the future.

## Methods

### Data collection

We used TCGAbiolinks package (v2.23.1) [29] to download somatic mutation (MUTECT2 version), transcriptome expression profile, miRNA expression profile, and TCGA-LUAD clinical data (The TCGA version is Hg38 (downloaded 2021-11-13). SNP6 GRCh38 served as reference information for copy number variation (CNV) data for GISTIC2 analysis, and the mapped probe file for CNV analysis data was retrieved from the TCGA-GDC database (https://portal.gdc.cancer.gov). The clinicopathological characteristics and predictive information of patients with LUAD, such as sex, age, and stage, were also retrieved (Table 5).

**Table 5:** Baseline clinical data

Data types	Sample numbers	Sample numbers of TP53-MUT	Sample numbers of TP53-WT
Somatic mutation (Mutect2)	568	278	289
Transcriptome expression Profile	585	249	260
miRNA expression Profile	567	249	260
Clinical data	510	247	255

### Mutational analysis

We used the maftools package (version 2.8.5) [30] to present the mutation panorama of LUAD and the mutant lollipop of *TP53* based on the somatic mutation data of LUAD. Based on the CNV data and other information about the cancer sample, the GISTIC tool module (version 2.0.23, default parameter) of the GenePattern website (https://cloud.genepattern.org/gp/pages/index.jsf) was used to view the missing and considerably amplified regions of the LUAD sample and the reference genome was GRCH38 [31].

### TMB and MSI analyses

Patients with LUAD were separated into TP53-MUT and TP53-WT groups based on their gene expression profiles to investigate the differences between them. The number of mutated bases per million bases in each tumor was calculated as the TMB. The TMB score was calculated for each LUAD sample as the total number of somatic mutations (including non-synonymous point mutations, insertions, and deletions)/target region size in mutations per Mb [32]. In addition, we used the R package deconstructSigs (v1.8.0) to determine the variance in the mutational signatures [13]. Microsatellites (MS) are short tandem repeats (STR) in the human genome, which include single nucleotide repeats, double nucleotide repeats, and higher nucleotide repeats. MSI is defined as an alteration in the length of the microsatellite that occurs in tumor tissue due to the insertion or deletion of a repeat unit and can be calculated using the number of insertions or deletions that occur in repeated sequences. The association between TP53-MUT and TP53-WT, TMB, and MSI was determined using MSIPRED [33]. To better understand the response of the TP53-MUT and TP53-WT groups to immunotherapy, we explored the differences in the expression of the immune checkpoints *LAG3*, *IDO1*, *PDCD1*, *CTLA4*, and *TIGIT*.

### Drug sensitivity and GSVA analyses

The LUAD cell line drug susceptibility dataset was downloaded from GDSC [34]. OncoPredict (v0.2) [35] was used to analyze the expression data of TP53-MUT and TP53-WT in patients with LUAD in the TCGA-LUAD dataset for drug sensitivity, and their susceptibilities to various LUAD treatments were compared.

GSVA is a nonparametric, unsupervised method used to calculate the enrichment score of a specific gene set in each sample [36, 37]. To study the biological variation between TP53-MUT and TP53-WT groups, we analyzed the differential expression of dysregulated pathways in these groups in the TCGA dataset using the R package GSVA (v1.40.1) [36, 37]. In addition, to calculate the normalized enrichment score (NES) of each sample in each pathway, we downloaded the reference gene sets “h.all.v7.4. symbols.gmt, and c2.cp.kegg.v7.4. symbols.gmt” in the MSigDB database (https://www.gsea-msigdb.org/gsea/msigdb) [36].

### Identification of differentially expressed genes (DEGs)

We downloaded TCGA-LUAD mutational data and divided the TCGA dataset into TP53-MUT and TP53-WT groups, to calculate DEGs between the two groups. The R package DESeq2 (v1.32.0) was used to perform variation analysis of TP53-MUT and TP53-WT. DEGs were set as follows: (logFC ≥ 1 or P < 0.05), the difference in upregulated expression was set as (logFC ≥ 1, P < 0.05), and the difference in downregulated expression was set as (logFC ≤ − 1, P < 0.05). We also divided DEGs into differentially expressed mRNAs, micro RNAs (miRNAs), and long non-coding RNAs (lncRNAs). Volcano plots were used to identify the differentially expressed mRNAs, miRNAs, and lncRNAs.

### Functional enrichment analysis

GO enrichment analysis is a general and functional method for large-scale functional enrichment analysis of genes across different dimensions and levels. It is typically conducted at three levels: biological process (BP), molecular function (MF), and cellular component (CC) [38]. KEGG is a popular database for storing data on genomes, biological pathways, illnesses, and medications [39–41]. The R package clusterProfiler (v4.0.5) [42] was used to identify the significantly enriched processes by GO functional annotation of DEGs and KEGG pathway enrichment analysis (Processes were considered significantly enriched at P < 0.05).

GSEA is a computational method used to determine whether a predefined set of genes exhibits statistical differences between two biological states; it is frequently used to evaluate changes in the activity of pathways and biological processes in expressed dataset samples [43]. To investigate the genetic differences in biological processes between the TP53-MUT and TP53-WT groups, we collected gene expression profiling data from patients in the TCGA-LUAD dataset and downloaded the reference gene sets "c5.go.v7.4. enttrez.gmt", "c2.cp.kegg.V7.4. entrez.gmt," and "H.ALL.V7.4. symbols.gmt" from the MSigDB database. We performed enrichment analysis of gene expression profiling data using the GSEA method included in the R package cluster Profiler. Statistical significance was set at P < 0.05.

### PPI and competing endogenous (CeRNA) networks

The STRING database searches for known proteins and predicts PPI [44]. In this study, the STRING database (https://string-db.org) [45] was used to construct a PPI network (default parameter) related to DEGa. Cytoscape (v3.8.2) was used to visualize the PPI network [46]. The tightly connected local regions in the PPI network may represent molecular complexes with specific biological functions. The MCODE network clustering algorithm can be used to mine protein complexes or corresponding functional modules from complex protein networks [47]. We extracted hub genes in the PPI subnetwork and those in the PPI subnetwork with an MCODE score greater than 10.

Single-stranded RNA molecules that are not encoded by endogenous genes are called miRNAs. They are approximately 19–25 NT in length and play a major role in biological evolution. miRNAs regulate the expression of target genes by participating in post-transcriptional regulation, playing an important role in tumorigenesis, biological development, organ formation, epigenetic regulation, and viral defense [48]. In other words, the network of miRNA regulation is extremely complicated, and a single miRNA may simultaneously affect several target genes [49]. LncRNA molecules regulate epigenetic processes, transcription, and post-transcription of protein-coding genes, but do not encode proteins [50]. Competing endogenous RNA (ceRNA) is a functional element that competes with binding genes to control the binding of RNA-coding genes. The ceRNA regulation network (ceRNA) is composed of mRNA, miRNA, and lncRNA. To analyze the relationship between miRNAs, lncRNAs and DEGs at the post-transcriptional stage, differentially expressed miRNAs in the miRNet database (https://www.mirnet.ca) and differentially expressed lncRNAs in the TCGA-LUAD database were collected and intersected to construct the network regulation of the mRNA-miRNA-lncRNA regulatory network. The R package Cytoscape was used to construct the mRNA-miRNA-lncRNA network [51].

### Immune infiltration analysis

The immune microenvironment is composed of the tumor, immune, stromal, and extracellular environments. ESTIMATE analysis is an algorithm for quantifying immunological activity (the amount of immune invasion) in tumor samples using gene expression data, which might reflect the number of gene features in the matrix and immune cells. The R package estimate (v1.0.13) was used to evaluate the stromal and immune cell contents in TCGA-LUAD [52]. To calculate the immune-associated scoring for input samples, we used the ESTIMATE database.

CIBERSORT is an algorithm based on linear support vector regression that deconvolves the expression matrix of immune cell subtypes using RNA-Seq data to estimate the abundance of immune cells in tissues [53]. We used the CIBERSORT algorithm to evaluate the proportion of the 22 immune cell subtypes in the immune microenvironment of TCGA-LUAD. Samples with accurate estimates of immune cell infiltration abundance were set using 1000 permutations (P ≤ 0.05). Based on Pearson’s correlation analysis, we calculated the correlation between the expression of characteristic genes and *TP53* in the prognostic model and 22 types of LUAD immune cells. We downloaded the expression data of infiltrating immune cells from CIBERSORTx (https://cibersortx.stanford.edu/). And then, based on the LM22 background gene in CIBERSORTx, we calculated the content of 22 immune cells in each patient to represent the infiltration level, selected the data with an immune cell enrichment score greater than zero, and then obtained and displayed the specific results of the immune cell infiltration abundance matrix. In addition, we used the R package IOBR (v0.99.9) [54] to calculate the immune infiltration results of xCell, EPIC, TIMER, CIBERSORTx, MCPcounter, QuanTIseq, and IPS, and pheatmap (v1.0.12) to display the heatmap, and the Wilcoxon test in the stats (v4.1.0) package to determine the significance of the difference between the TP53 MUT group and the TP53 WT group.

In addition, immune-related genes were downloaded from the ImmPort database (https://www.immport.org) [55] and cross-linked with differential mRNA to analyze the relationship between cross-linked immune genes and *TP53* mutations. We also examined the association of *HLA* genes (both typical *HLA* genes, such as *HLA-DPA* and *HLA-DRB* and atypical *HLA* genes, such as *HLA-Z* and *TAP2*) recorded in the IMGTLAHLA database(http://hla.alleles.org/genes/index.html) with TP53-MUT and TP53-WT [56].

### Prognostic model

We used the clinical and mutation information to construct a prognostic model. First, we performed univariate and multivariate Cox analyses according to age, sex, clinical stage, and tumor stage in patients with LUAD harboring mutant *TP53*. We then calculated the independent predictive power of clinicopathological features for overall survival (OS), incorporated the corresponding indicators into the model, and created a nomogram.

### Statistical analysis

All statistical analyses were performed using the R software (https://www.r-project.org/, R 4.1.0). The false-discovery rate (FDR) was corrected to P values using the Benjamini-Hochberg (BH) method, to reduce the false-positive rate. The Mann–Whitney U test (Wilcoxon rank-sum test) was used to analyze the difference between non-normally distributed variables when comparing two sets of continuous variables. The R package Survival (v3.2.11) [57] was used to perform survival analysis, Kaplan–Meier analysis to determine survival differences, log-rank test to show the survival time differences, and uni- and multi-variate Cox regression analyses to identify independent prognostic factors. All P values in this study were two-sided. P ≤ 0.05 was considered statistically significant.

## Supplementary Information

**Additional file 1.** **SF1.** Workflow. TCGA: The Cancer Genome Atlas; LUAD: Lung Adenocarcinoma; GISTIC: Genomic Identification of Significant Targets in Cancer; CNV: Copy Number Variations; TMB: Tumor Mutation Burden; MSI: Microsatellite Instability; PPI: Protein-Protein Interaction; URA: Univariate Regression Analysis; MRA: Multivariate Regression Analysis KM: Kaplan Meier.

**Additional file 2.** **ST1.** Information of TP53 TOP10 mutation frequency. The top 10 information items of TP53 mutations stored by mutation frequency in TP53 include mutation location, mutation type, codon variation, number of mutation samples, and mutation frequency.

**Additional file 3.** **SF4.** Immunohistochemical staining of p53 protein was in normal and tumor tissues. In the HPA database. Immunohistochemical staining of p53 protein was lighter in normal tissues than in tumor tissues.

**Additional file 4.** **SF3.** Immune infiltration results of ESITMATE, xCell, EPIC, TIMER, CIBERSORTx, MCPcounter, quanTIseq, and IPS. The columns in the heatmap represent samples; yellow represents TP53 MUT samples, and green represents TP53 ET samples. The rows represent different cells in each immune infiltration analysis, and the content between the parentheses indicates the significance of the difference in immune infiltration between the TP53 MUT and TP53 WT groups (*: p <= 0.05; **: p <= 0.01; ***: p <= 0.001; ****: p <= 0.0001). The heatmap matrix depicts the immune infiltration levels derived from various techniques. In ESTIMATE, red represents high immune infiltration levels, whereas blue represents low immune infiltration levels. In xCell, yellow represents high immune infiltration levels, whereas dark purple represents low immune infiltration levels. In EPIC, red represents high immune infiltration levels, whereas blue represents low immune infiltration levels. In TIMER, light green represents high immune infiltration levels, whereas dark green represents low immune infiltration levels. In CIBERSORTx, light red represents high immune infiltration levels whereas dark red represents low immune infiltration levels. In MCPcounter, yellow represents high immune infiltration levels, whereas deep purple represents low immune infiltration levels. In quanTIseq, yellow represents high immune infiltration levels, whereas dark blue represents low immune infiltration levels. In IPS, yellow represents high immune infiltration levels, whereas darkred represents low immune infiltration levels.

## Data Availability

The datasets for this study can be found in the following website: https://portal.gdc.cancer.gov, https://cloud.genepattern.org/gp/pages/index.jsf, https://www.gsea-msigdb.org/gsea/msigdb, https://string-db.org, https://www.mirnet.ca, https://www.immport.org, http://hla.alleles.org/genes/index.html. All data generated or analyzed during this study are available upon reasonable request from the corresponding author.
