# Supplementary figures and images for: Integrative analysis of TP53 mutations in lung adenocarcinoma for immunotherapies and prognosis
Source: BMC Bioinformatics. 2023 Apr 18;24:155. doi: 10.1186/s12859-023-05268-2 (PMC10114340; doi:10.1186/s12859-023-05268-2)

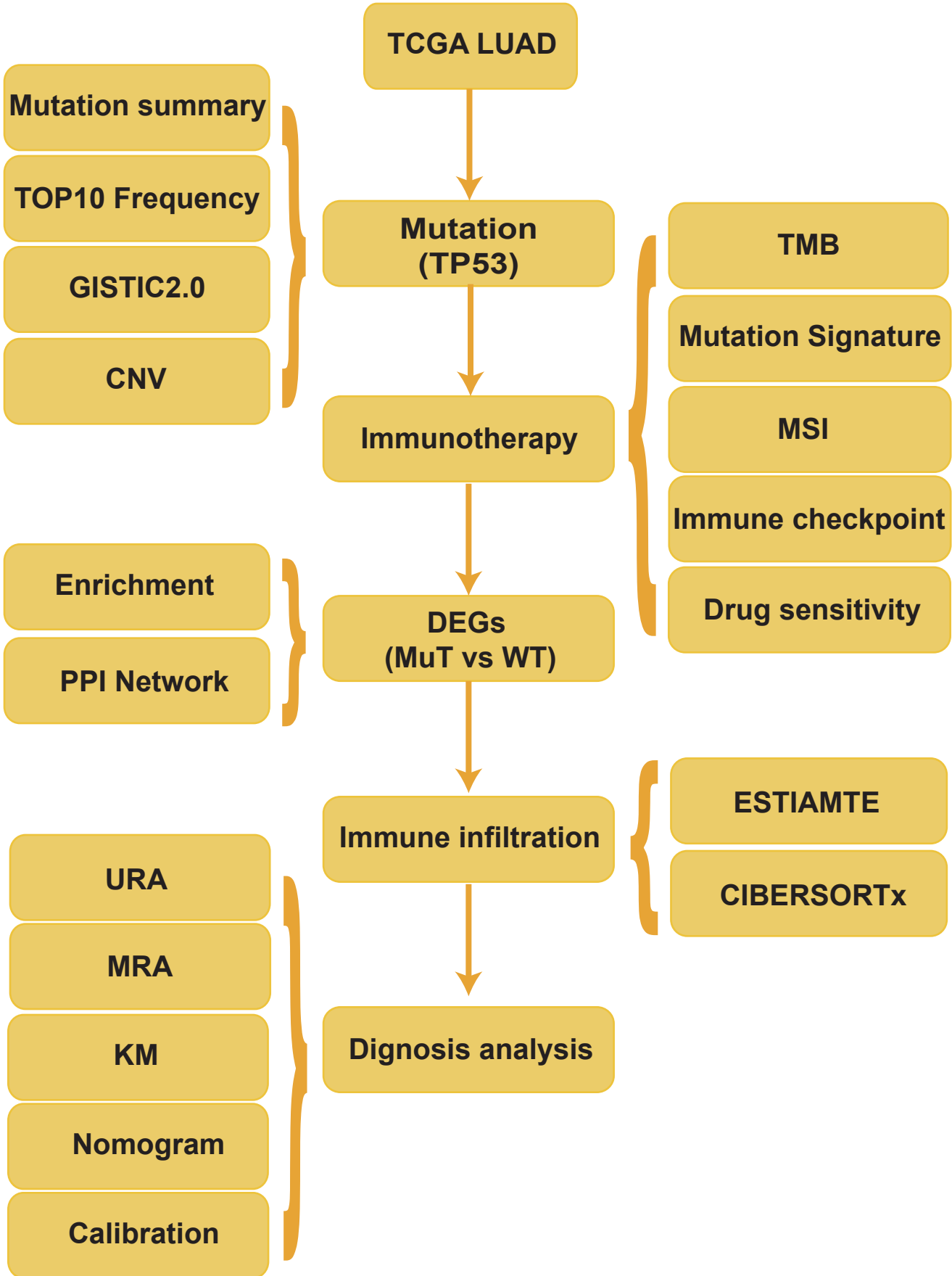

Supplement: Supplementary file 1 — Additional file 1. SF1. Workflow. TCGA: The Cancer Genome Atlas; LUAD: Lung Adenocarcinoma; GISTIC: Genomic Identification of Significant Targets in Cancer; CNV: Copy Number Variations; TMB: Tumor Mutation Burden; MSI: Microsatellite Instability; PPI: Protein-Protein Interaction; URA: Univariate Regression Analysis; MRA: Multivariate Regression Analysis KM: Kaplan Meier. [file 12859_2023_5268_MOESM1_ESM.pdf]

normal

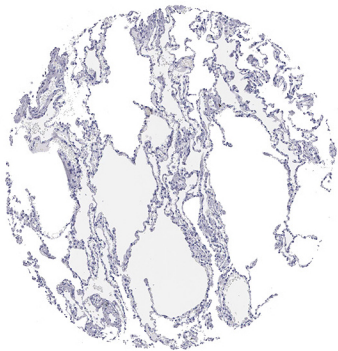

TP53-HPA2417

tumor

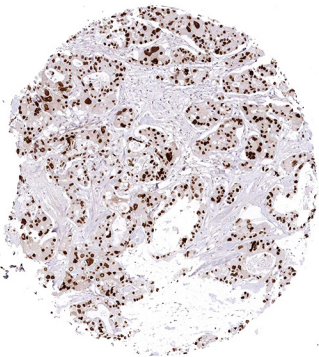

TP53-HPA2393

tumor

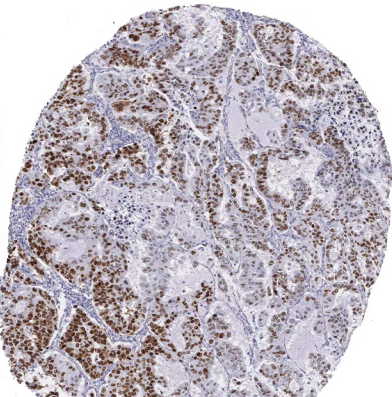

TP53-HPA1847

tumor

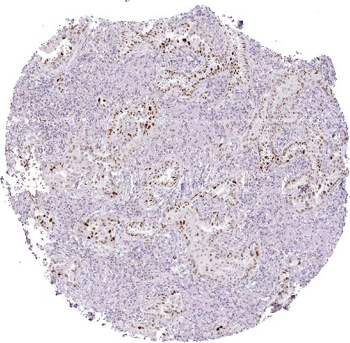

TP53-HPA2777

Supplement: Supplementary file 3 — Additional file 3. SF4. Immunohistochemical staining of p53 protein was in normal and tumor tissues. In the HPA database. Immunohistochemical staining of p53 protein was lighter in normal tissues than in tumor tissues. [file 12859_2023_5268_MOESM3_ESM.pdf]

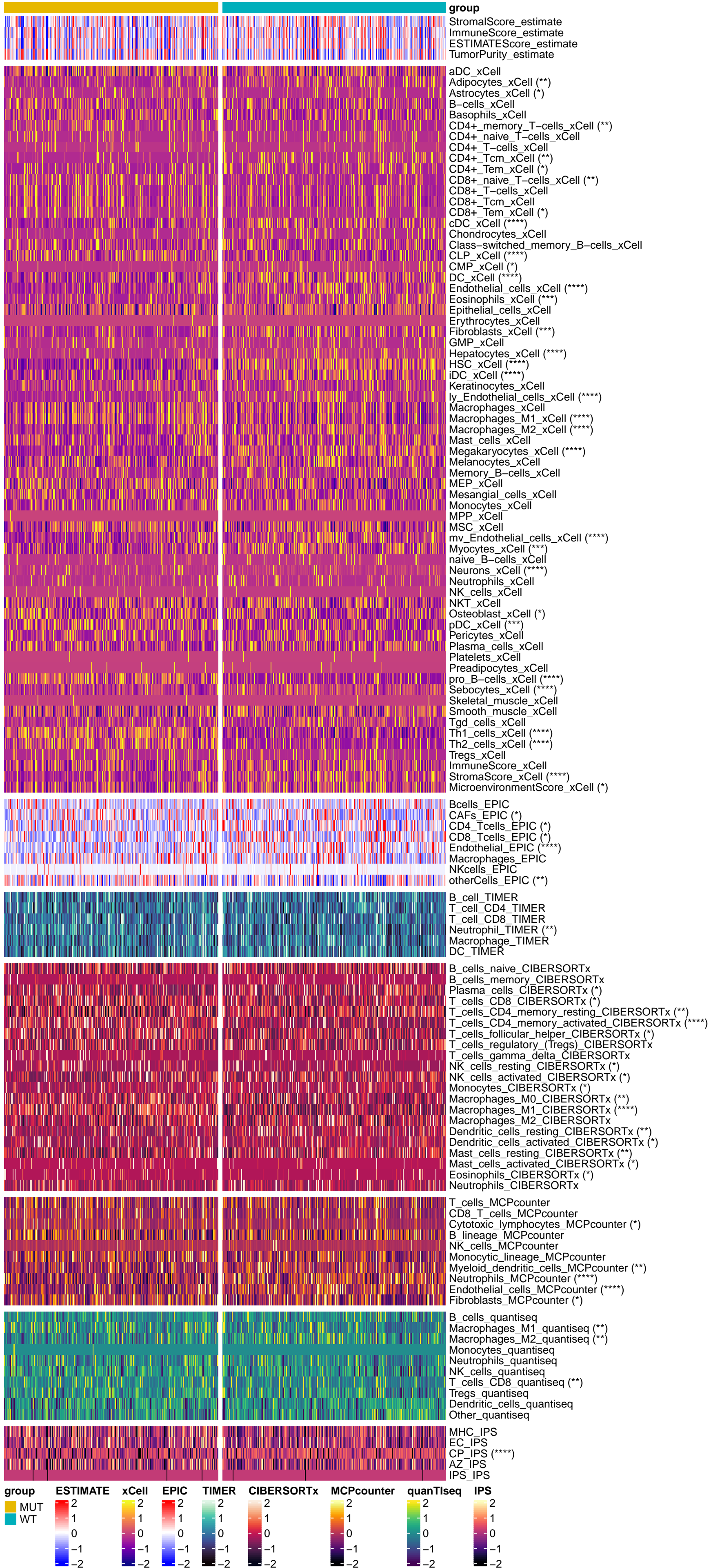

Supplement: Supplementary file 4 — Additional file 4. SF3. Immune infiltration results of ESITMATE, xCell, EPIC, TIMER, CIBERSORTx, MCPcounter, quanTIseq, and IPS. The columns in the heatmap represent samples; yellow represents TP53 MUT samples, and green represents TP53 ET samples. The rows represent different cells in each immune infiltration analysis, and the content between the parentheses indicates the significance of the difference in immune infiltration between the TP53 MUT and TP53 WT groups (*: p <= 0.05; **: p <= 0.01; ***: p <= 0.001; ****: p <= 0.0001). The heatmap matrix depicts the immune infiltration levels derived from various techniques. In ESTIMATE, red represents high immune infiltration levels, whereas blue represents low immune infiltration levels. In xCell, yellow represents high immune infiltration levels, whereas dark purple represents low immune infiltration levels. In EPIC, red represents high immune infiltration levels, whereas blue represents low immune infiltration levels. In TIMER, light green represents high immune infiltration levels, whereas dark green represents low immune infiltration levels. In CIBERSORTx, light red represents high immune infiltration levels whereas dark red represents low immune infiltration levels. In MCPcounter, yellow represents high immune infiltration levels, whereas deep purple represents low immune infiltration levels. In quanTIseq, yellow represents high immune infiltration levels, whereas dark blue represents low immune infiltration levels. In IPS, yellow represents high immune infiltration levels, whereas darkred represents low immune infiltration levels. [file 12859_2023_5268_MOESM4_ESM.pdf]
